# Extensive Diversity of *Streptococcus pyogenes* in a Remote Human Population Reflects Global-Scale Transmission Rather than Localised Diversification

**DOI:** 10.1371/journal.pone.0073851

**Published:** 2013-09-16

**Authors:** Rebecca J. Towers, Jonathan R. Carapetis, Bart J. Currie, Mark R. Davies, Mark J. Walker, Gordon Dougan, Philip M. Giffard

**Affiliations:** 1 Menzies School of Health Research, Division of Global and Tropical Health, Casuarina, Northern Territory, Australia; 2 Telethon Institute for Child Health Research, Centre for Child Health Research, University of Western Australia, West Perth, Western Australia, Australia; 3 Australian Infectious Diseases Research Centre, University of Queensland, St. Lucia, Queensland, Australia; 4 The Wellcome Trust Sanger Institute, The Wellcome Trust Genome Campus, Hinxton, Cambridge, United Kingdom; St. Petersburg Pasteur Institute, Russian Federation

## Abstract

The Indigenous population of the Northern Territory of Australia (NT) suffers from a very high burden of *Streptococcus pyogenes* disease, including cardiac and renal sequelae. The aim of this study was to determine if *S. pyogenes* isolated from this population represent NT endemic strains, or conversely reflect strains with global distribution. *emm* sequence typing data were used to select 460 *S. pyogenes* isolates representing NT *S. pyogenes* diversity from 1987–2008. These isolates were genotyped using either multilocus sequence typing (MLST) or a high resolution melting-based MLST surrogate (Minim typing). These data were combined with MLST data from other studies on NT *S. pyogenes* to yield a set of 731 MLST or Minim typed isolates for analysis. goeBURST analysis of MLST allelic profiles and neighbour-joining trees of the MLST allele sequences revealed that a large proportion of the known global *S. pyogenes* MLST-defined diversity has now been found in the NT. Specifically, fully sequence typed NT isolates encompass 19% of known *S. pyogenes* STs and 43% of known *S. pyogenes* MLST alleles. These analyses provided no evidence for major NT-endemic strains, with many STs and MLST alleles shared between the NT and the rest of the world. The relationship between the number of known Minim types, and the probability that a Minim type identified in a calendar year would be novel was determined. This revealed that Minim types typically persist in the NT for >1 year, and indicate that the majority of NT Minim types have been identified. This study revealed that many diverse *S. pyogenes* strains exhibit global scale mobility that extends to isolated populations. The burden of *S. pyogenes* disease in the NT is unlikely to be due to the nature of NT *S. pyogenes* strains, but is rather a function of social and living conditions.

## Introduction

The bacterium *Streptococcus pyogenes* is an important human pathogen [1]. It is a common cause of pharyngitis (“strep throat”) and skin infections. It can also cause invasive and systemic infections that may be life threatening A distinctive aspect of *S. pyogenes* infections is the rare but significant occurrence of immune related sequelae. Acute rheumatic fever (ARF) is an inflammatory condition that primarily affects the joints and the cardiac muscle [2]. Repeated episodes of ARF can lead to rheumatic heart disease (RHD) [1], [3], which is associated with severe and sometime fatal heart valve damage. Acute post-streptococcal glomerulonephritis (APSGN) results from deposition of antibody-amtigen complexes in the glomerula basement membrane, with consequent temporary impairment of renal function. [4], [5] ARF and RHD are largely regarded as sequels of pharyngitis, and APSGN as a sequel of skin infection. ARF, RHD and APSGN are all rare in developed countries and are associated with high burdens of infection, and sub-optimal provision of health care [1].

There is no vaccine available against *S. pyogenes* infections. The diverse, immuno-dominant, antiphagocytic surface located M protein can elicit M type specific immunity. However because of the diversity of the M protein, this immunity can have limited coverage of strains. Current vaccine development initiatives are focussed either on multiple valency [6], or using conserved domain(s) in the M proteins do elicity proader immunity [7].

The Australian Northern Territory (NT) is extraordinarily sparsely populated. Two hundred and thirty-three thousand people occupy 1.4 million km^2^, which is more than the areas of Spain, Portugal, France and Great Britain combined. Thirty percent of this population identify as Aboriginal, about half of whom live in small Aboriginal communities, many of which are in very remote locations. The NT Indigenous population is disadvantaged with respect to most measures of health and socio-economic well-being. *S. pyogenes-*associated pyodermas (skin sores) are very common in some communities, particularly in children, often secondary to scabies infestation [8], [9].

The north of the NT is mainly monsoonal tropical savannah. In the Indigenous population of this zone, *S. pyogenes* pharyngitis appears rare [10]. Despite this, this population has one of the highest burdens of ARF and RHD in the world [2], which challenges the dogma that *S. pyogenes* skin infections do not play a role in these pathologies [10], [11]. Paradoxically, in the southern region of the NT, which is arid with low humidity, pyoderma rates appear lower than in the northern region, pharyngitis is more common, but the incidence of acute rheumatic fever and the prevalence of rheumatic heart disease are similar to the tropical north [11]. Post-streptococcal glomerulonephritis is also frequently seen in the NT, with epidemics seen in the northern region and sporadic cases in the south [12], [13]. There is evidence that streptococcal infections in childhood are a contributing factor to the very high prevalence of renal failure in Indigenous adults [12].

*S. pyogenes* isolates from NT Aboriginal communities have been characterized by *emm* sequence analysis [14], [15], [16], and in some instances by multilocus sequence typing (MLST) [17], [18]. The emerging picture is that there is considerable genetic diversity. Here we address the question as to whether this diversity reflects a long history of *S. pyogenes* evolution within northern Australia prior to European settlement, or conversely is a consequence of a more recent ingress of multiple global *S. pyogenes* strains into the NT Indigenous population, as has been suggested by Currie [19]. This question is relevant with regards to both susceptibility of the local population to infection for control of *S. pyogenes* disease by vaccination using sequence-type specific vaccines [6].

## Methods

### Ethics Statement

This study made use of pure bacterial cultures that had previously been collected in a variety of research projects involving human subjects and from diagnostic service providers, and stored frozen. This study involved no human experimentation or use of previously unpublished human clinical data.

The *S. pyogenes* isolates used were all derived from the NT, and stored at the Menzies School of Health Research in Darwin, Australia. They were collected between 1987 and 2008 and were either clinical isolates obtained from the Royal Darwin Hospital, or collected in the course of research projects carried out in Aboriginal communities *emm* sequence subtype data are available for 1,732 of these isolates, and these encompass 104 *emm*ST and 142 *emm* sequence subtypes.

Minim typing was performed as previously described [18]. Minim typing is based on MLST. [18], [20], [21], [22]. The *S. pyogenes* Minim typing system encompasses high resolution melting (HRM) analysis of 10 stretches of DNA internal to the fragments used for MLST. Each fragment encompasses a single nucleotide polymorphism (SNP) that is one part of a set derived from the *S. pyogenes* MLST database on the basis of maximization of the Simpsons Index of Diversity (*D*). Other SNPs in the fragments confer additional resolving power. Minim data is interpreted by converting the HRM curves to inferred G+C content. A current key for converting between *S. pyogenes* Minim data and MLST data is provided as supplementary data (Table S1). Minim types are referred to as Melting Types (MelTs).,

MLST was performed as described by Enright and co-workers [23], and the database accessed at http://spyogenes.mlst.net/. Inference of population structures from MLST profiles using goeBURST was performed as previously described by Francisco and co-workers ( [24] and the software was accessed at http://goeburst.phyloviz.net/.

## Results

Four hundred and sixty isolates representing *S. pyogenes* diversity in the NT were assembled by selecting one isolate from each year for each *emm* sequence subtype. These were all subjected to Minim typing. Where new MelTs, or new combinations of *emm*ST and MelT were observed, MLST was performed. These data were combined with MLST data previously reported [17], [18], and MLST data extracted from 70 genome sequences currently under analysis, the final result being 731 NT isolates with associated Minim and/or MLST data. (Table S2). This included MLST data for 400 isolates and Minim typing data for 491 isolates. The results from the 156 isolates typed by both methods displayed 100% concordance. In total, 121 MLST STs and 128 MelTs were identified, including 45 new STs (ST572-ST617). The details of 200 isolates were deposited in the *S. pyogenes* MLST database at http://spyogenes.mlst.net/.

**Figure 1:**
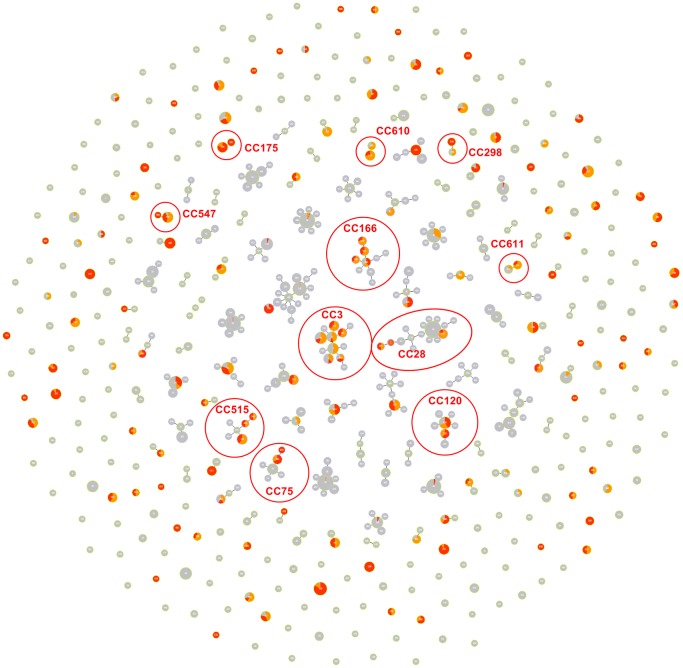
GoeBURST highlighting NT strains (red = MLST data; orange = MLST inferred from Minim typing and emmST). Grey circles or sectors are STs thus far identified only outside the NT. Clonal complexes with NT isolates in two or more STs are circled. Each spot in the GoeBURST diagram is labelled with an ST number. These may be visualised by zooming in.

The diversity of NT *S. pyogenes* MLST allele profiles within the global context was visualised by goeBURST analysis ( [24], http://goeburst.phyloviz.net/) (Fig 1, Table S3). As shown in Fig 1, MLST STs found in the NT (shown in red and orange) are distributed throughout the global *S. pyogenes* structure, with no evidence for NT specific clonal complexes. All groups of >2 STs linked by single locus variant (SLV) relationships were composed either of NT and non-NT isolates or entirely of non-NT isolates, with the mixed (NT plus non-NT isolates) groups being numerically dominant. Therefore, we were unable to demonstrate that any major strain or clonal complex has evolved extensively and specifically within the NT, and conclude that the diversity observed in the NT S. pyogenes population is not due to diversification of endemic strains.

MLST allele sequence comparisons lead to a similar conclusion. Neighbour-joining trees of all known alleles at each of the seven MLST loci were assembled (Fig. S1), and there is no evidence for clustering of MLST alleles observed in the NT *S. pyogenes* isolates (“NT alleles”). Alleles found in the NT represent 43% of all the alleles in the MLST database. Fifty-four (23%) of NT alleles have not been found elsewhere, but these are not clustered and 47 differ at a single base and 6 differ at two bases from alleles found outside Australia. The single remaining NT-specific allele, *murI*_73, appears to be a chimera in part derived from *Streptococcus dysgalactiae* subsp. *equisimilis. murI*_73 was found in two STs: ST572 and ST588, which are single locus variants (SLVs) of each other. ST572 is an SLV of ST166, which has been found in the NT, the UK and The Gambia. ST572 and ST588 represent just three of the 731 Menzies isolates with associated MLST and/or Minim typing data, and the relevant *emm*STs, *emm77* (ST572 and ST588), and *stKNB1* (ST572) represent just 15 of the 1732 Menzies isolates that have been subjected to *emm* analysis. Therefore, the unusual *murI-73* allele may represent an example of recombination that has occurred within the NT, but it is not a marker of a numerically significant NT-specific strain.

**Figure 2:**
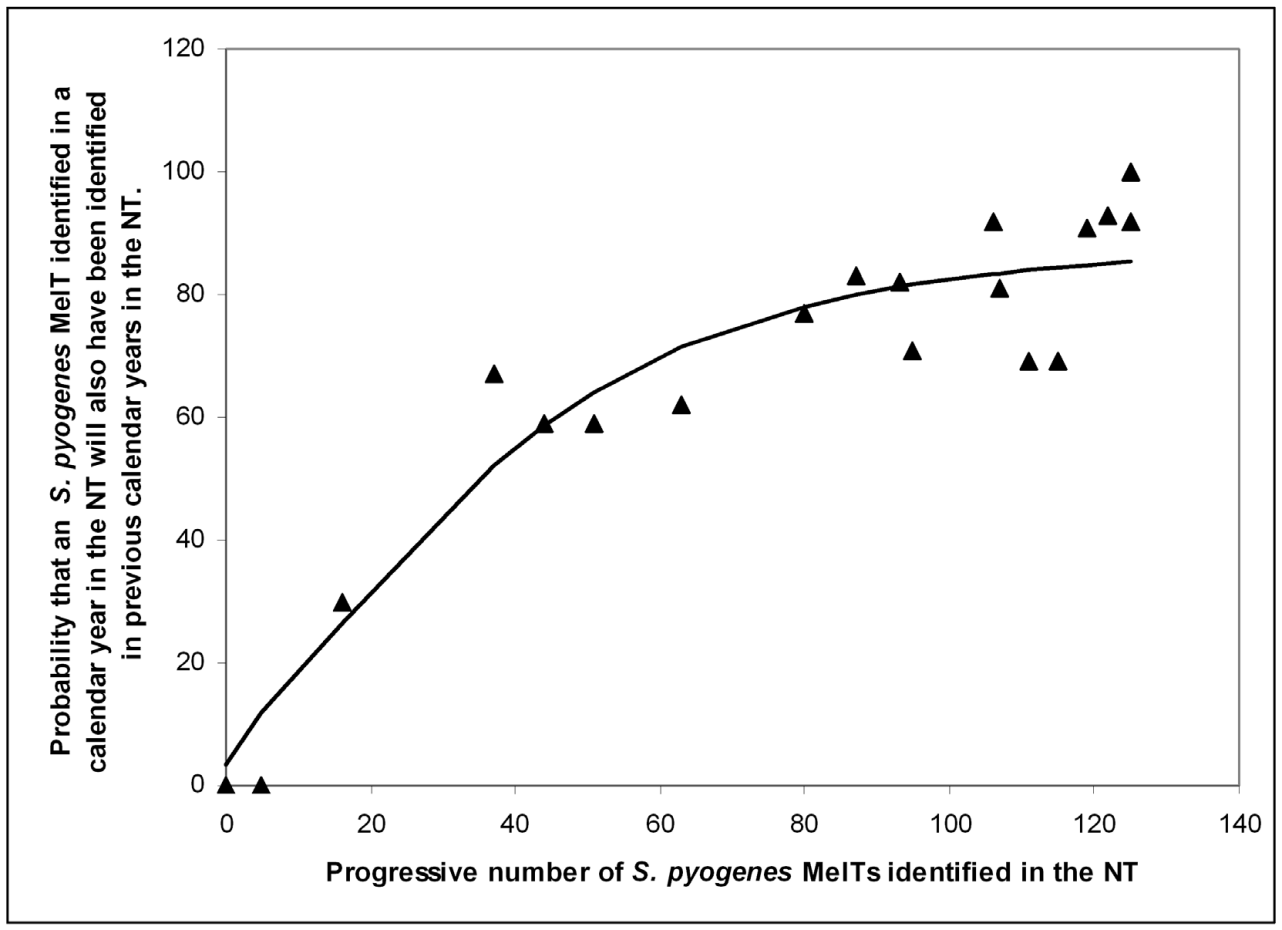
The relationship between the cumulative total of already discovered MelTs, and the probability that a MelT identified in any given calendar year will be not be novel. The line of best fit was calculated using a regression constrained to asymptote to a value ≤100%. Its formula is y = 88.4((exp(0.582√×−3.18)/(1+exp(0.582√×−3.18)).

In order to estimate the depth of sampling of the NT *S. pyogenes* population, we determined the relationship between the proportion of MelTs identified in any given year that had been identified in previous years, and the cumulative total number of MelTs identified in the years preceding each year in question. Figure 2 demonstrates that the probability that a MelT identified in a calendar year will have been previously identified rises markedly as a function of the cumulative total of known MelTs. This indicates that a significant proportion of the total NT *S. pyogenes* diversity has been identified.

## Discussion

We have shown that *S. pyogenes* isolated from the sparse and often isolated human population of the NT represents a remarkably large subset of the known global *S. pyogene*s population structure and diversity, as defined by MLST. We could find no evidence for major *S. pyogenes* strains endemic to the NT. Previous studies of NT *S. pyogenes* have revealed considerable diversity, and it has been noted that similar emmSTs (sequence types based on a portion of the M protein encoding *emm* gene) are found both in the NT and elsewhere [16], [25], [26]. In contrast, a previous MLST-based study of NT *S. pyogenes* indicated that there are many NT-specific *S. pyogenes* STs. [17]. However our results show that this is not the case, and that the results of McGregor and co-workers [17] may be explained by their smaller sample size of NT isolates, and the smaller size of the *S. pyogenes* MLST database at the time. Our results are similar to what was found with paediatric *S. pyogenes* isolates from Western Nepal, where 120 isolates encompassed 51 STs and 45 *emm* types, many of which had been identified elsewhere [27]. Nine STs have been identified as occurring both in Western Nepal and the NT: ST109, ST119, ST120, ST140, ST181, ST267, ST289, ST297 and ST348, thus reinforcing the global mobility of *S. pyogenes*. While the presence of *S. pyogenes* disease in the NT Indigenous population prior to European colonization cannot be completely excluded, this study provided no evidence for this and is consistent with the notion that the burden of *S. pyogenes* disease in the NT Indigenous population post-dates European colonization [19].

There was sampling bias in our study, in that the 460 isolates were selected for Minim typing on the basis of representing the range of the *emm* subtype diversity in our collection. It is conceivable that major core-genome defined *S. pyogenes* strain(s) unique to the NT have been missed. However, in this and other studies [17], [18], 731 (42%) of the 1732 *emm* subtyped isolates from the Menzies collection have now been subject to Minim typing and/or MLST. For a numerically significant NT endemic strain to remain undetected, all isolates of that strain would have to possess the same *emm* subtype as isolate(s) already analysed by MLST and or Minim typing, and also have not been analysed by MLST and or Minim typing This is highly unlikely. Furthermore, a recent study of NT *S. pyogenes* showed that the cumulative proportion of isolates increases smoothly with the number of *emm* types, indicating a lack of dominant strains [16]. While we cannot disprove the existence of *S. pyogenes* strains unique to the NT, such strains are at most rare.

The relationship between the number of known MelTs and the probability that a MelT identified in a calendar year will be new, suggests that the numerically significant *S. pyogenes* strains in the NT have been identified. The line of best fit in Fig 2 indicates that at the termination of sampling, ∼80% of MelTs identified in a calendar year had been identified in previous calendar years. Therefore, a large proportion of the detected strains persist in the NT for >1 year. We were unable to estimate the rate or dynamics of strain turnover with greater accuracy, because the intensity and locations of sampling varied from year to year. However, recent outbreaks of streptococcal glomeronephritis in the NT, some associated with known internationally circulating nephritic strains, show that strain turnover has occurred within the period of sample collection [13].

These findings support the view that the high burden of *S. pyogenes* disease in the NT Indigenous population relates to socioeconomic factors. It is well documented that remote Indigenous communities have a much higher point prevalence of streptococcal infection than the non-Indigenous population,

We therefore conclude that these communities which exhibit an extremely high brurden of streptococcal disease are very efficient at sampling form the global *S. pyogenes* population. This is most likely a function of socioeconomic factors and limited access to health care which long been known to contribute to streptococcal transmission and disease [10]. Public health interventions that target the prevalence of skin infections are likely to have the greatest impact, at least in the tropical north of the NT. While there is good evidence for outbreaks of acute post-streptococcal glomerulonephritis in the NT [6], there is no discernable evidence in the NT for an association between particular *S. pyogenes* strains and rheumatic heart disease. In contrast to this, outbreaks of rheumatic fever that have been observed in the United States [28], [29]. Our results suggest that the burden of rheumatic heart disease in the NT Indigenous population is a simple function of the magnitude of the burden of superficial *S. pyogenes* infection, possibly modulated by a genetic component of host susceptibility [30]. There is therefore little justification for interventions that target any specific *S. pyogenes* strains, with the exception of known nephritogenic strains. Consistent with this, current Northern Territory Government mandated public health interventions target eradication of nephritogenic clones with widespread use of intramuscular benzathine penicillin (http://www.health.nt.gov.au/library/scripts/objectifyMedia.aspx?file=pdf/10/84.pdf&siteID=1&str_title=Acute Post-Streptococcal Glomerulonephritis.pdf.).

The negative consequence of the diversity of NT *S. pyogenes* upon the likely efficacy in the NT of an M-protein based vaccine targeting only a subset of *emm* types in the NT [6] has already been noted [16]. However, the significance of our study extends beyond the NT by providing really good evidence for the rapid global mobility of many *S. aureus* strains. This suggests that many, if not all human populations, including those in developed countries, may come in contact with considerable *S. pyogenes* diversity over short time scales. This, in turn has the potential to facilitate replacement of strains targeted by a vaccine by strains not targeted by the vaccine.

In conclusion, the circumstances of the NT and the diverse population structure iof NT *S. pyogenes* clearly indicate that addressing social determinants and/or the use of an *S. pyogenes* vaccine with broad specificity are the most promising strategies for reducing the burden of *S. pyogenes* disease in the NT Indigenous population,.

## Supporting Information

Table S1 **Key used to translate between Minim and MLST data.** This is a minor update of the key provided to support the description of *S. pyogenes* Minim typing [18], and is used in the same way.
(XLS)

Table S2 **Isolates included in this study, with** ***emm*** **sequence subtype and MLST and/or Minim typing data.**
(XLS)

Table S3 **MLST and/or Minim typing data, together with geographic information, used to generate** **Fig 1** **.**
(XLS)

Figure S1 **Neighbour joining trees of** ***S. pyogenes*** **MLST loci.** Red highlighted alleles have been found in the Australian Northern Territory only. Yellow highlighted alleles have been found in the Northern Territory and elsewhere. Non-highlighted alleles have not been found in the Northern Territory.
(PPT)
