# Supplementary figures and images for: Extensive Diversity of Streptococcus pyogenes in a Remote Human Population Reflects Global-Scale Transmission Rather than Localised Diversification
Source: PLoS One. 2013 Sep 16;8(9):e73851. doi: 10.1371/journal.pone.0073851 (PMC3774777; doi:10.1371/journal.pone.0073851)

## Slide 1
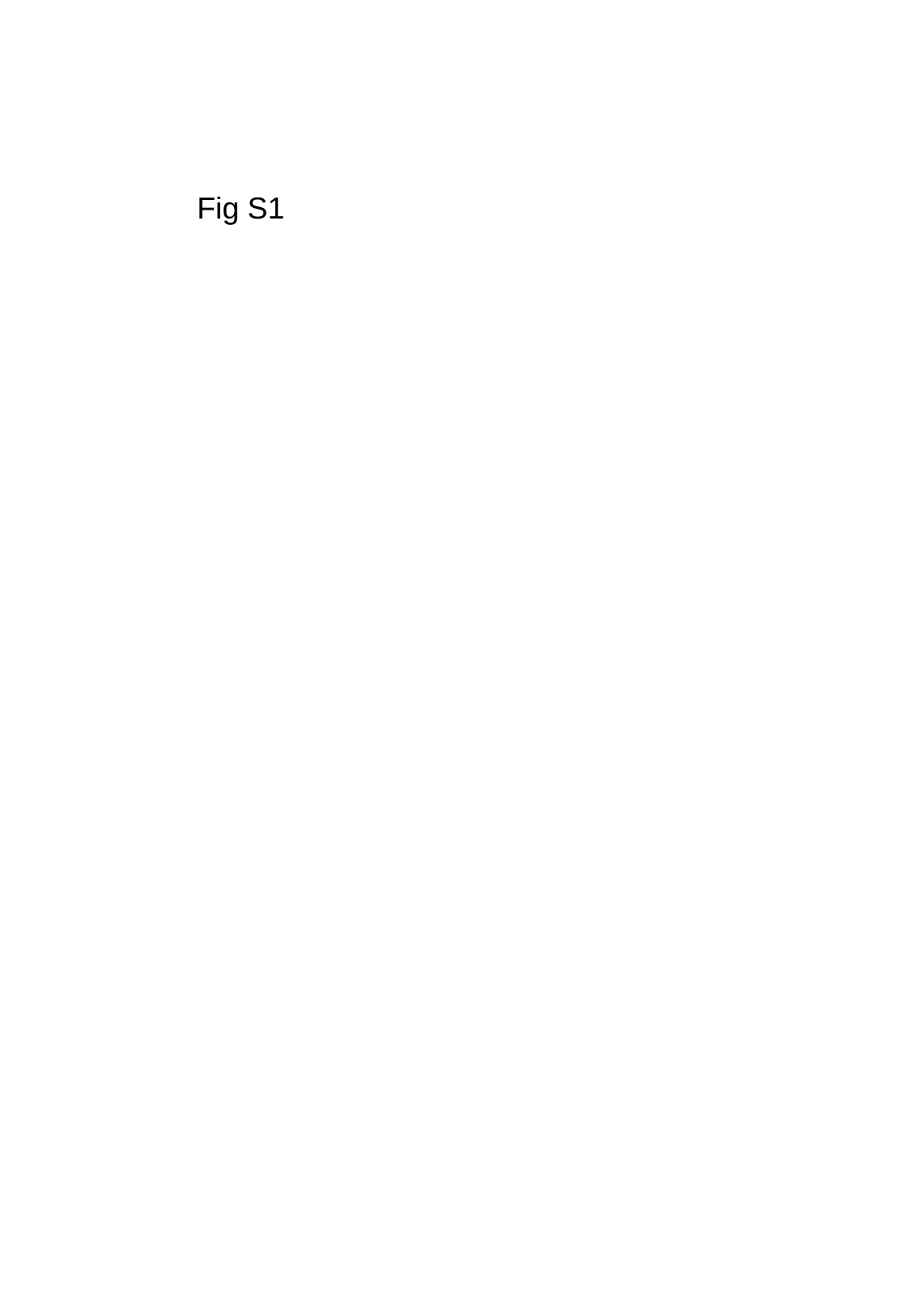

Fig S1

## Slide 2
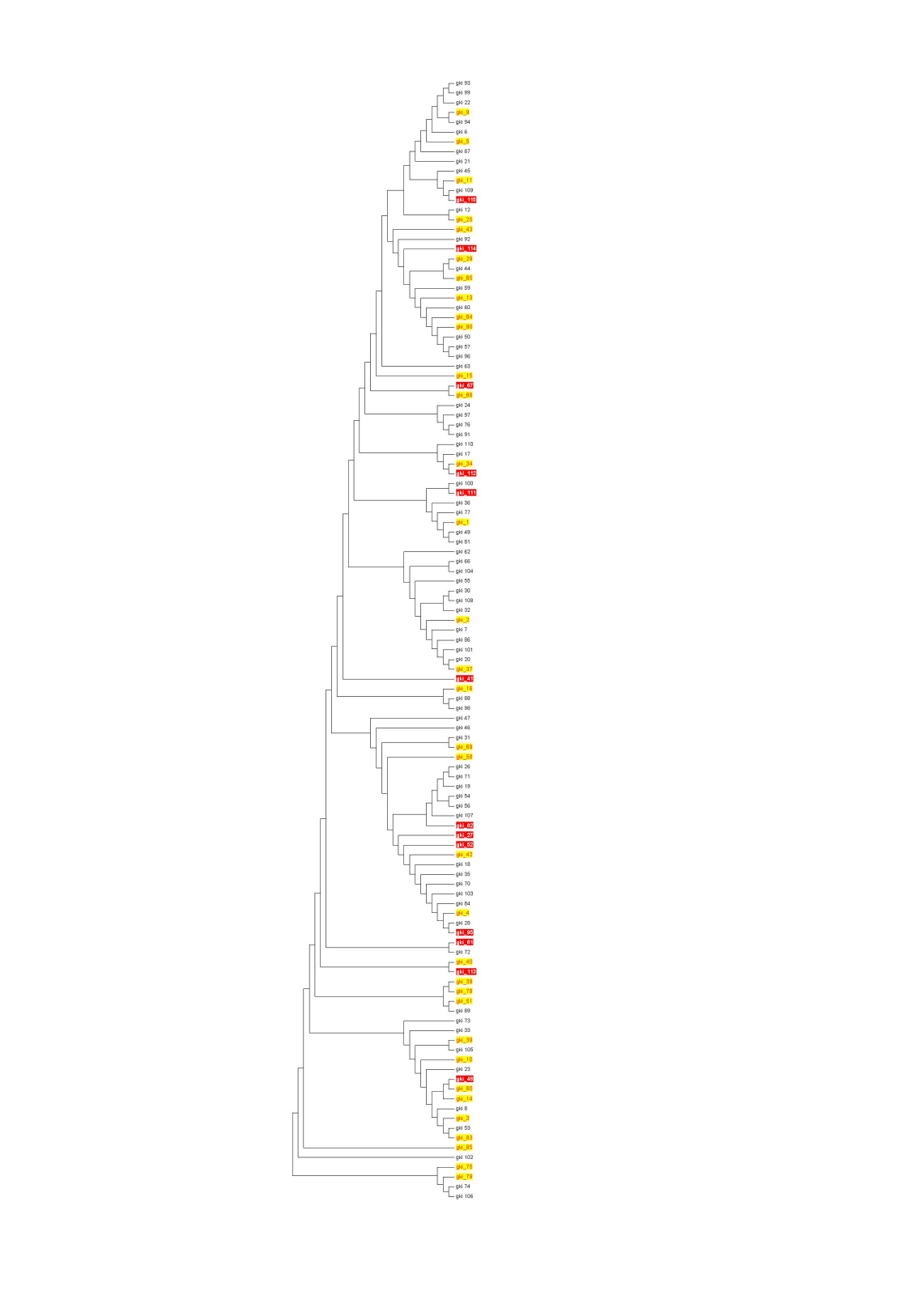

## Slide 3
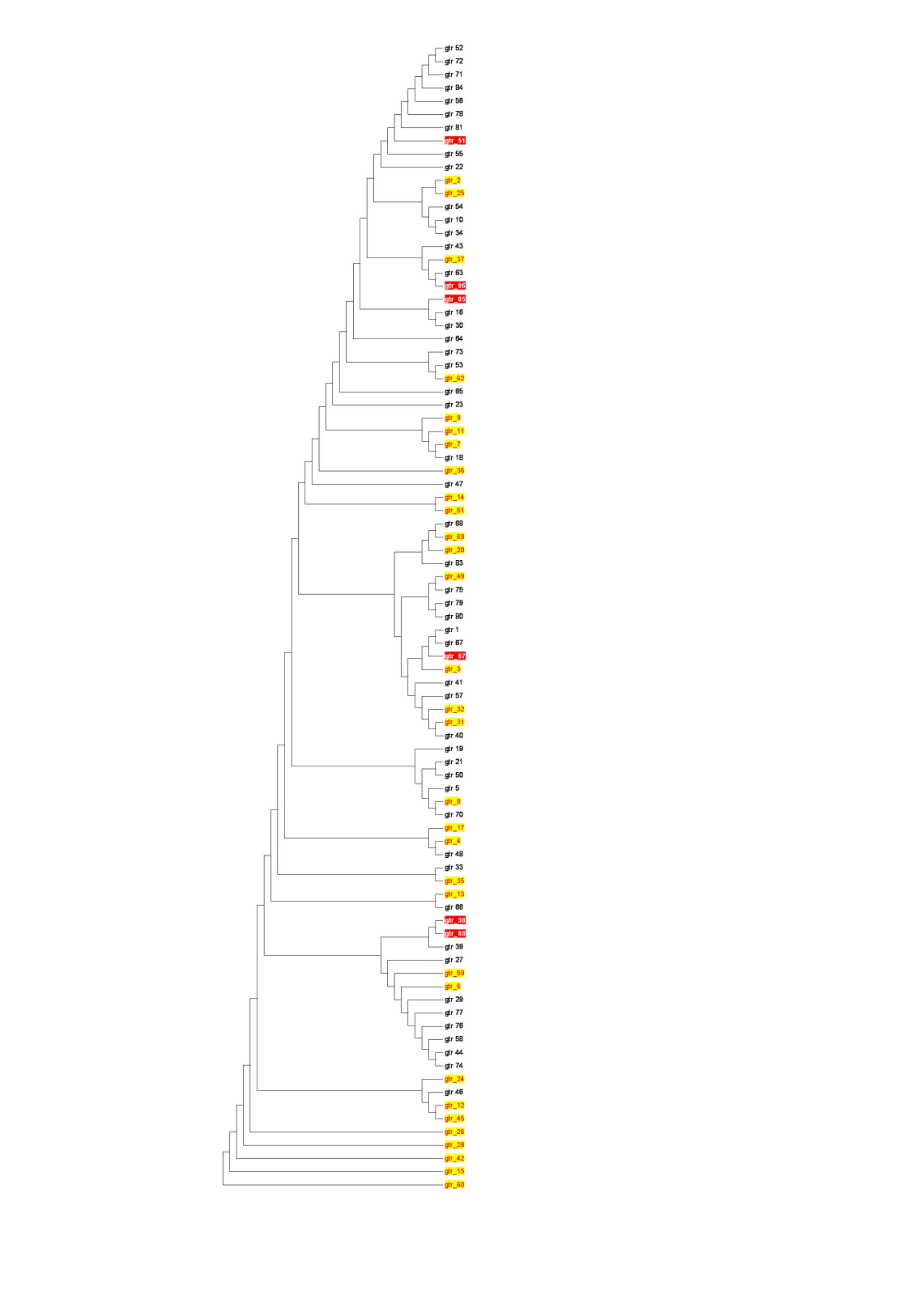

## Slide 4
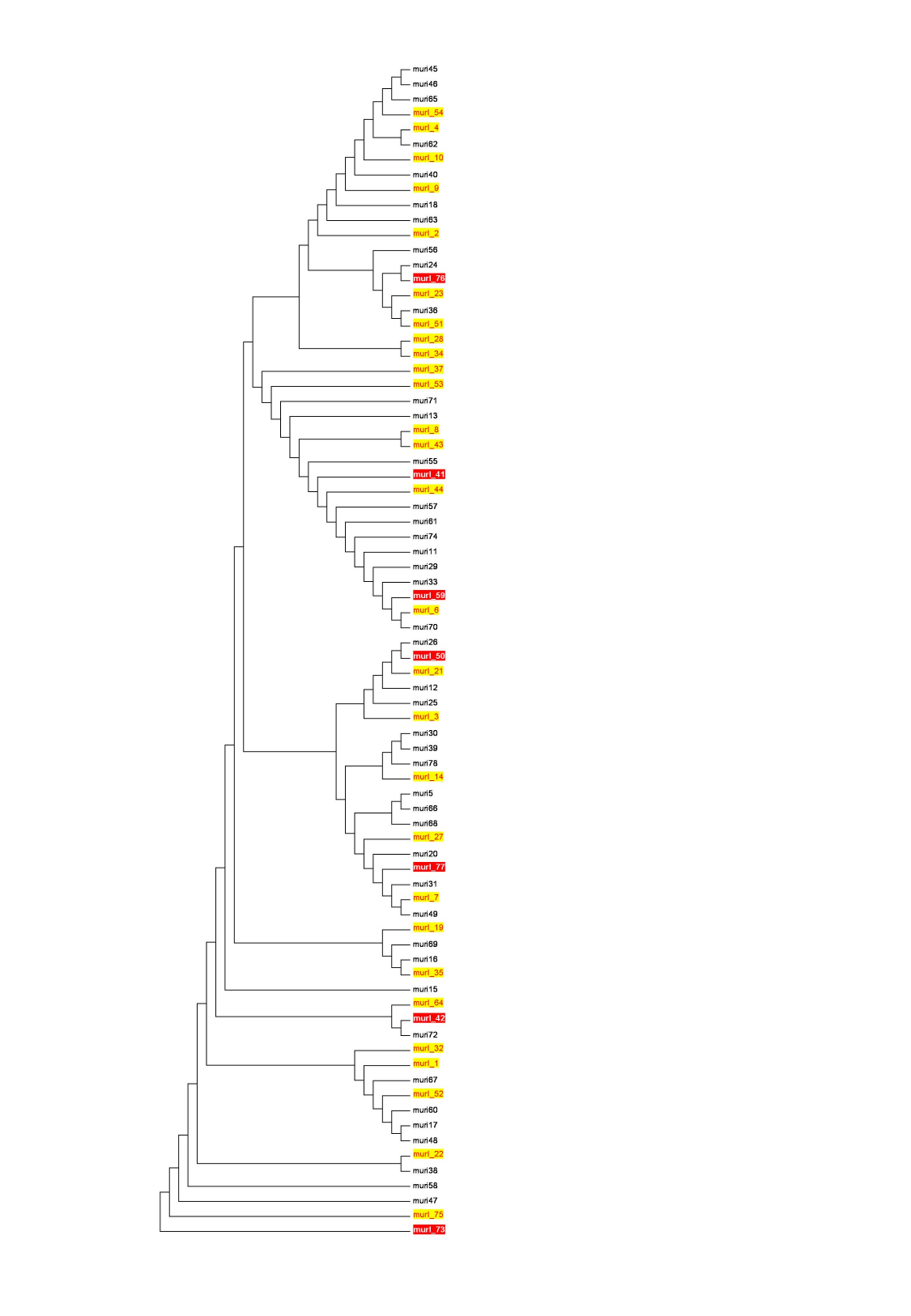

## Slide 5
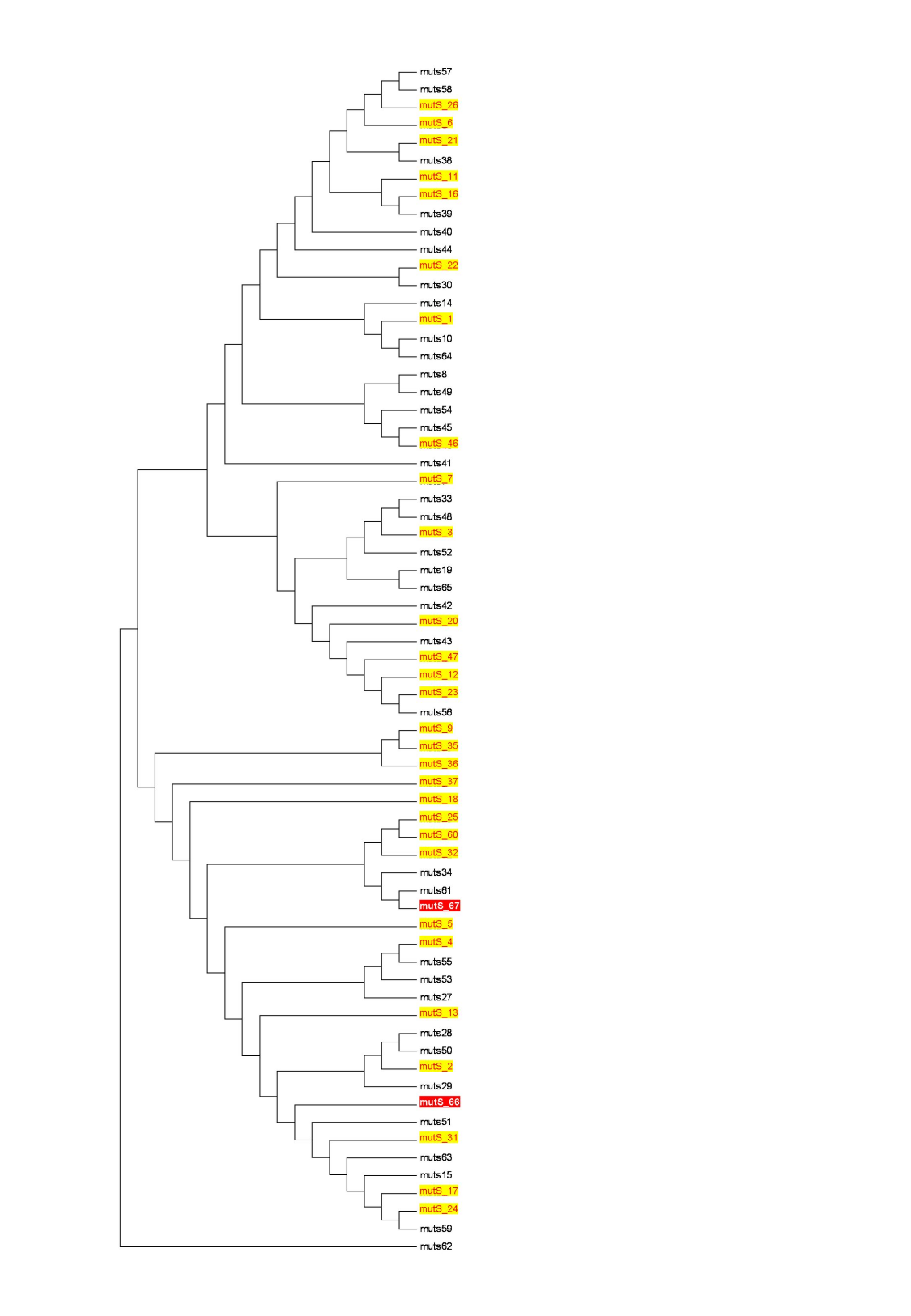

## Slide 6
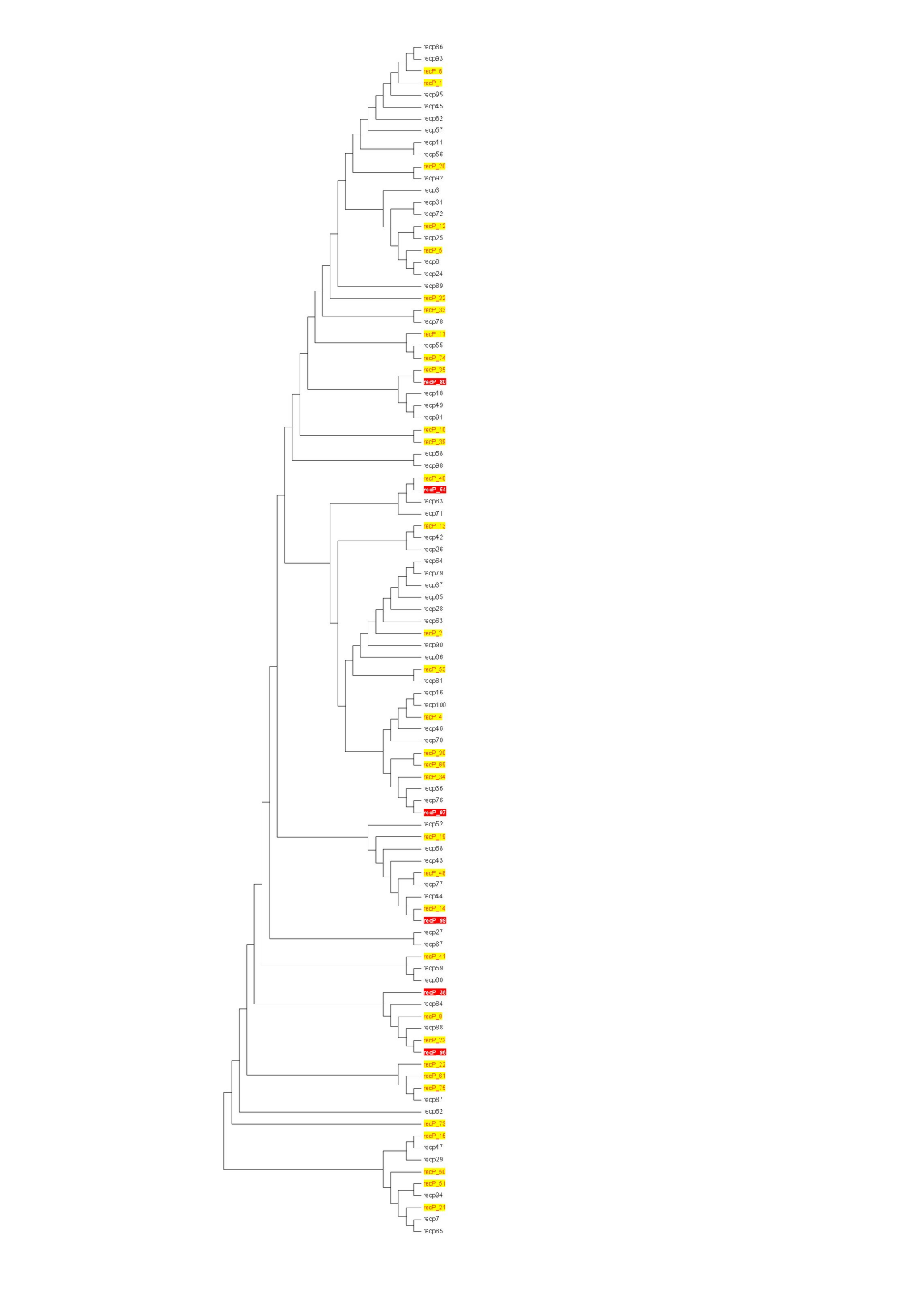

## Slide 7
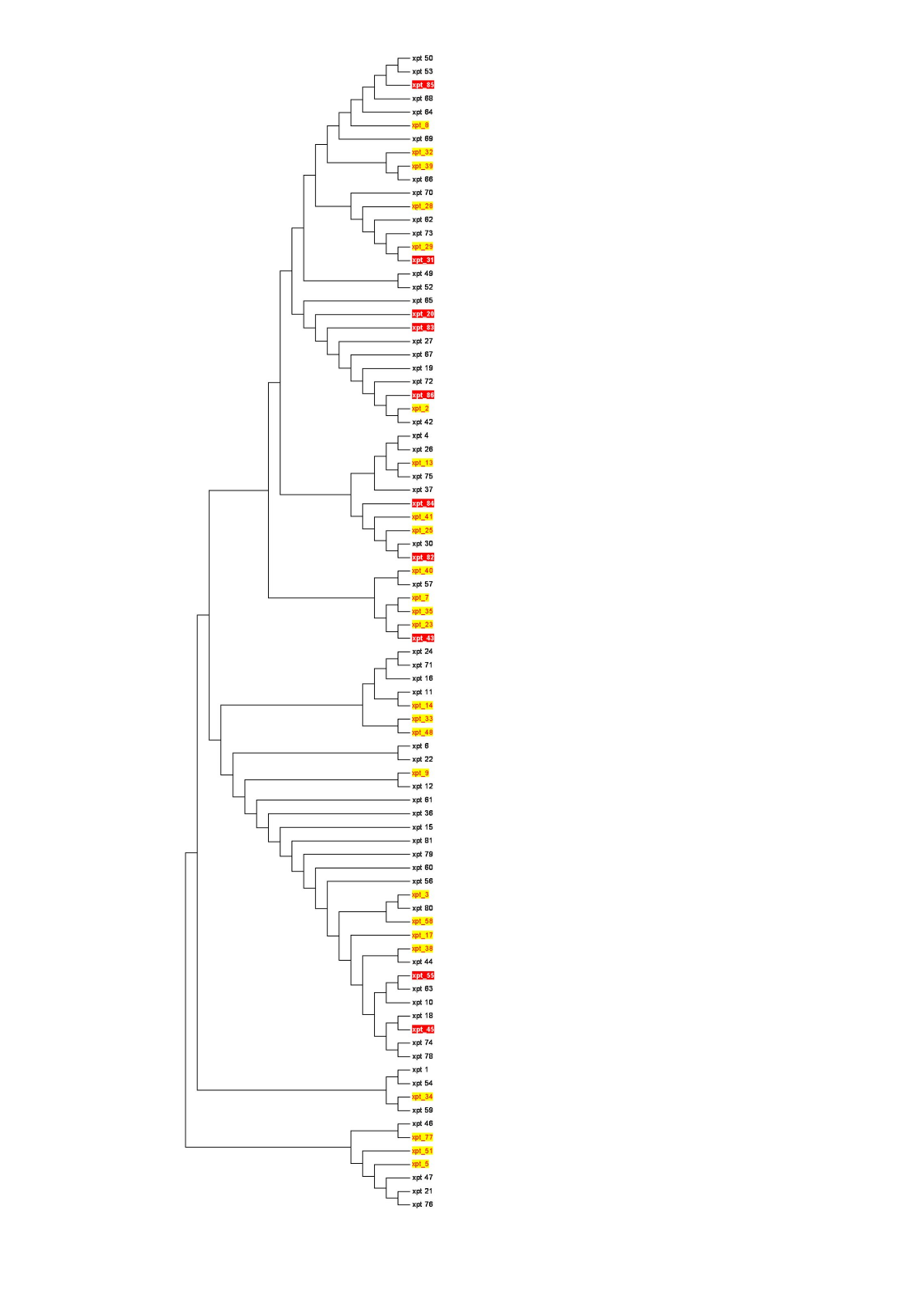

## Slide 8
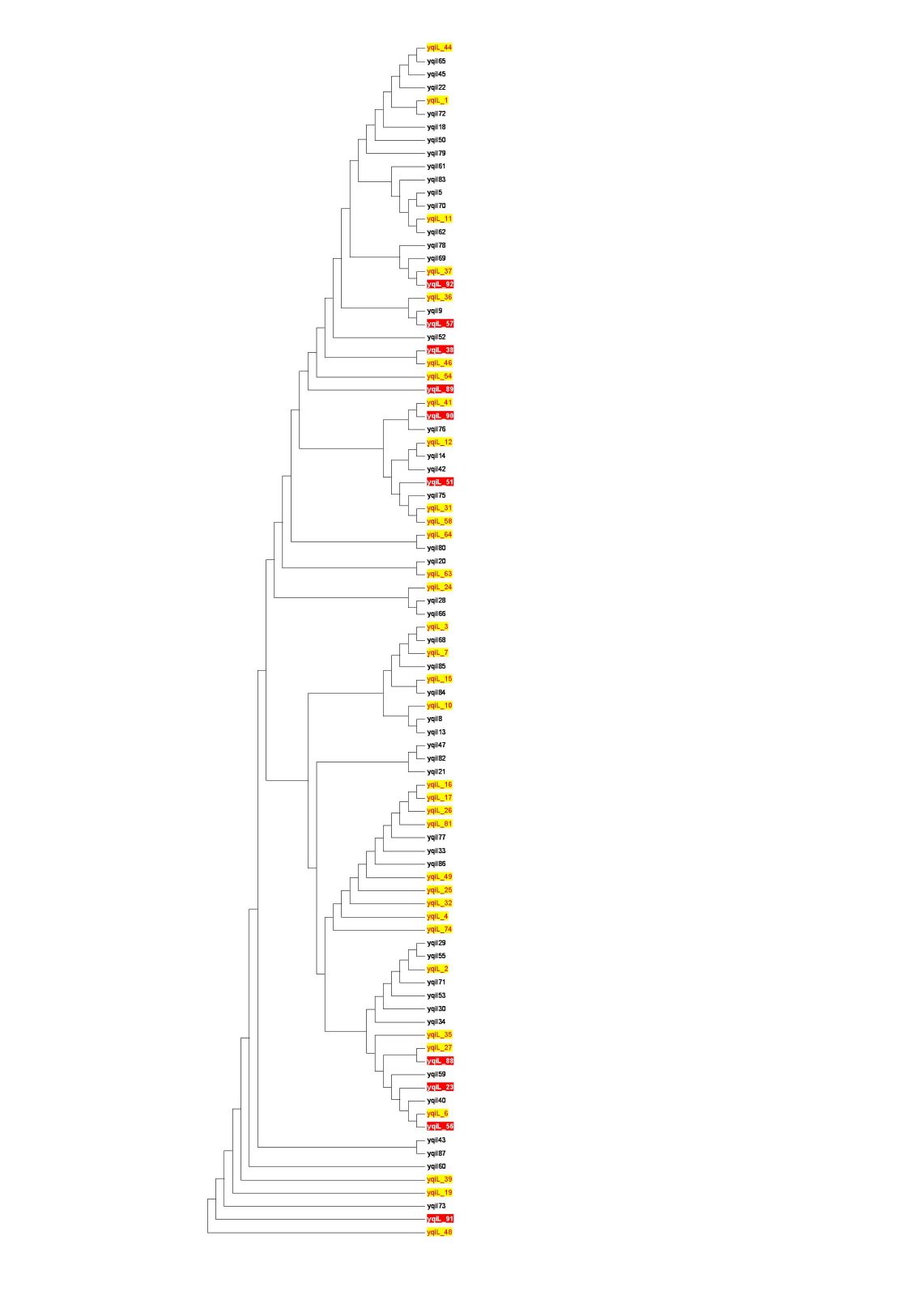

Supplement: Figure S1 — Neighbour joining trees of S. pyogenes MLST loci. Red highlighted alleles have been found in the Australian Northern Territory only. Yellow highlighted alleles have been found in the Northern Territory and elsewhere. Non-highlighted alleles have not been found in the Northern Territory. (PPT) [file pone.0073851.s004.ppt]
